# Changes in Antidepressant Absorption After Metabolic Bariatric Surgery

**DOI:** 10.1007/s11695-025-08025-x

**Published:** 2025-07-04

**Authors:** Lucas Sabatella, Azucena Aldaz Pastor, Manuel Fortún Landecho Acha, Rafael Moncada Durruti, Daniel Aliseda Jover, Nuria Blanco Asensio, Victor Valentí Azcárate

**Affiliations:** Clínica Universidad de Navarra, Pamplona, Spain

**Keywords:** Metabolic bariatric surgery, Antidepressants, Drug metabolism, Therapeutic range

## Abstract

**Introduction:**

Metabolic bariatric surgery (MBS), particularly Roux-en-Y gastric bypass (RYGB), is one of the most effective long-term intervention for weight loss, but its hypoabsortive nature may affect drug metabolism.

**Methods:**

A retrospective longitudinal study with intra-individual comparisons was conducted on patients who underwent RYGB at Clínica Universidad de Navarra between 2014 and 2019 at our institution and were on antidepressant treatment before and after surgery. Apparent oral clearance (CL/F), concentration/dose ratio (CDR), and weight loss parameters were compared pre- and post-surgery. Measurements were taken at the time of surgery (M1), 1 month after surgery (M2), and between 6 and 15 months after surgery (M3).

**Results:**

Fourteen patients (10 females) with a mean age of 48.92 years and a mean baseline *BMI* of 37.32 kg/m^2^ were included in the study. They were being treated with fluoxetine (*n* = 6), duloxetine (*n* = 2), bupropion (*n* = 2), sertraline (*n* = 1), clobazam (*n* = 1), topiramate (*n* = 1), and aripiprazole (*n* = 1). Patients who were within the therapeutic range for their medications prior to surgery remained within that range postoperatively. The fluoxetine + D-fluoxetine concentrations and CDR significantly varied between M1 and M2, with a *p*-value of 0.022, and an inverse association between *BMI* and D-fluoxetine CDR was observed (*p* = 0.004).

**Conclusions:**

These findings suggest, in a small cohort, that chronic use of antidepressants does not require major changes in the management of patients undergoing MBS. Two distinct absorption patterns were identified for different antidepressants after surgery, highlighting the potential influence of metabolic pathways and enzymatic activity. The inverse association between D-fluoxetine CDR and *BMI* may be linked to changes in CYP enzyme function following MBS.

## Introduction

Obesity is a multifaceted chronic disease. According to the World Health Organization, 2.5 billion adults were overweight and 890 million had obesity in 2022 [1, 2].

Among weight-loss interventions, MBS is the most effective long-term strategy. RYGB remains as gold-standard procedure due to its combined restrictive and hypoabsorptive mechanism, but this same hypoabsorption raises concerns about drug metabolism and long-term complications [3–6].

Among patients with obesity, a high prevalence of mood disorders such as depression or anxiety has been observed, raising important questions about the relationship between obesity, mental health, and treatment with antidepressant drugs [7]. The interaction between antidepressant drugs and hypoabsorptive surgeries adds an additional layer of complexity to the management of obesity and its associated medical problems. Epidemiologically, there is evidence that the use of mood-disorder drugs does not decrease after RYGB [8]. In addition, a small excess risk of suicide associated with MBS has been described [9].

The available evidence on pharmacokinetic changes after RYGB is limited, with studies including only a small number of patients and follow-up periods that, in most cases, do not exceed 1 year postoperatively [10–17]. This knowledge gap is particularly concerning given that obesity is frequently associated with psychiatric disorders, and while some patients experience improvement after surgery, others do not. The lack of pharmacokinetic data in this context creates uncertainty among clinicians managing these patients, raising concerns about the effectiveness and safety of psychiatric medications after RYGB.

In this context, the present project aims to elucidate pharmacokinetic changes in patients with medication-dependent depression following RYGB.

## Material and Methods

A retrospective, longitudinal study was carried out on a prospectively collected database with intra-individual comparisons over time (2 measurements at least, one pre-surgery, and one post-surgery).

Patients undergoing laparoscopic gastric bypass at the Clínica Universidad de Navarra, Pamplona, Navarra, Spain, between 2014 and 2019, who met the following inclusion criteria were included: age over 18 years, having informed consent on the transfer of biological samples to the Biobank of the Clínica Universidad de Navarra for its pharmacokinetic analysis (the relevant ethical, legal, and technical procedures were followed) and receiving antidepressants both before and after surgery, and having confirmed adherence to the treatment. Since patients will act as their own control, they were screened if they had at least two blood samples for analysis, one before surgery and one after surgery. Thirteen out of the fourteen patients had at least three measurements, which were categorized as M1 (before surgery), M2 (1 month after surgery), and M3 (between 6 and 15 months after surgery).

Antidepressant drugs levels were analyzed in the Clinical Pharmacokinetics Unit of the Pharmacy Service by UPLC-MS/MS chromatography, using the analysis kit from ChromeSystem (MassTox® TDM Series A Parameter Set Antidepressants 1/Extended).

The following variables were collected: identification variables (medical record number); anthropometric variables including height (cm), weight (kg), body mass index (BMI), air displacement plethysmography with BOD-POD [18] and abdominal circumference collected before and after surgery on the different measurement dates; demographic variables (age and sex), treatment (type of drug, dose and frequency); and pharmacokinetic variables including baseline concentration (C0) of the drug in mcg/mL or ng/mL, apparent oral clearance (CL/F) in L/h and mL/h/kg, and weight-normalized concentration/dose ratio (CDR).

The apparent oral clearance (CL/F) and the CDR has been selected as the comparative pharmacokinetic measure between the pre-surgery and post-surgery situations, as these are considered as the main population pharmacokinetic parameters to describe the disposition of drugs and when assessing the impact of different factors, like surgery, on the pharmacokinetics in neuro-psychopharmacotherapy [19].

The *CL*/*F* was estimated according to Eqs. (1) and (2):

1 $$CL/F\;(L/h)=\lbrack dose/day\;(mg/day\;)\times1\;day/24\;h\rbrack/\lbrack C0\;(mcg/mL)\rbrack$$

2 $$CL/F\;(\mathrm{mL}/\mathrm h/\mathrm{kg})=\lbrack\mathrm{dose}/\mathrm{day}\;(\mathrm{mg}/\mathrm{kg}/\mathrm{day})\times1\;\mathrm{day}/24\;\mathrm h\rbrack/\lbrack\mathrm C0\;(\mathrm{mcg}/\mathrm{mL})\rbrack\times1\;\mathrm L/1000\;\mathrm{mL}\times1000/60$$

The *CDR* was estimated according to Eq. (3):

3 $$CDR\;(\mathrm{ng}/\mathrm{mL}\times\mathrm{mg})=\lbrack\mathrm C0\;(\mathrm{ng}/\mathrm{mL})\rbrack/\lbrack\mathrm{dose}/\mathrm{day}\;(\mathrm{mg}/\mathrm{day})\rbrack$$

The therapeutic ranges used to assess each patient’s drug levels were those established by Hiemke et al. in the *Consensus Guidelines for Therapeutic Drug Monitoring in Neuropsychopharmacology* [19], which serve as the gold standard for interpreting plasma concentrations and concentration-to-dose ratios of neuropsychiatric drugs.

In our Center, the use of fluoxetine as the antidepressant of choice in patients with obesity is standard practice due to its impact on weight loss and its safety profile. Consequently, the analysis of the results was divided into two groups: fluoxetine and other antidepressants.

All statistical analyses were performed using the Stata v.12 (StataCorp, College Station, TX, USA). The results were presented as *mean* (standard deviation, *SD*) for continuous variables and proportions for qualitative ones. The Student’s *t*-test for paired samples or the Wilcoxon test were performed depending on the normality of the variables studied, using *p* < 0.05 as the significance criterion. The Shapiro–Wilk test was performed to analyze the normality.

To evaluate the relationship between the concentration-to-dose ratio (*CDR*) of D-fluoxetine and BMI, while accounting for longitudinal variations and intra-individual comparisons, a linear mixed-effects model was employed. This approach allowed us to model repeated measurements within individuals and to assess the influence of time on pharmacokinetic changes. The model included *CDR* of D-fluoxetine as the dependent variable, with BMI and time (as a continuous variable) as fixed effects, *p* < 0.05 was set as the significance limit. To account for the correlation between repeated measures within the same patient, a random intercept for each individual was included, allowing for subject-specific baseline variations. The covariance structure was set as unstructured to allow for flexible correlation patterns between measurements. The model was fitted using maximum likelihood estimation (*MLE*). To visually represent the model predictions, we generated an adjusted margins plot using the “margins*”* and “marginsplot” commands in Stata.This allowed us to illustrate the estimated effect of *BMI* on *CDR* at different time points, adjusting for intra-individual variability.

## Results

Fourteen patients (10 females) were included in the study, with a mean age of 48.92 years (*SD* 2.32) and a mean *BMI* at baseline of 37.32 kg/m^2^ (*SD* 1.1). The total weight loss (*TWL*) between M1 and M3 was 28.41 (*SD* 2.69). Table 1 includes the anthropometric and demographic variables throughout the three measurements taken M1, M2, and M3.

**Table 1:** Demographics

	M1	M2	M3
Sex (M/F)	4/10		
Age	52.3 (4)		
*TWL* (%)		11.3 (3.3)	28.4 (9.4)
Weight (kg)	101.2 (3.1)	90 (2.7)	69.5 (4.2)
*BMI* (kg/m^2^)	36.9 (1.8)	32.5 (1.7)	25.2 (1.7)
Total fat (kg)	49.2 (2.9)	41.1 (3.6)	22.6 (5.6)
Waist circumference	120.3 (3.7)	111.8 (3.7)	91.8 (4.6)

*TWL*: Total weight loss; *BMI*: Body mass index; *Kg*: Kilograms; Mean (sd).

The results of pharmacokinetic variables across the study are shown in Table 2. It should be noted that twelve out fourteen patients were within the therapeutic range of their respective medications at all times; the other two patients were not in it before surgery. There was no change in the prescribed treatments that the patients were receiving between the preoperative and postoperative periods, except for the introduction of dietary supplements. Additionally, none of the other drugs was modified during the study period. It was also confirmed that none of the medications the patients were receiving acted as documented enzyme inducers or inhibitors.

**Table 2:** Pharmacokinetic changes of the drugs studied

PATIENT	DRUG	DOSE (mg/24 h)			*CONC* (ng/mL)				*CL* (mL/min)			*CDR* (ng/mL × mg)			
		M1	M2	M3	M1	M2	M3		M1	M2	M3	M1		M2	M3
1	Fluoxetine	20	20	20	95	122	168		217.01	127.42	195.61	4.75		6.1	4.2
	D-fluoxetine				64	109	142		146.19	113.84	165.34	3.2		5.45	3.55
	D-F/F				0.67	0.89	0.85		0.67	0.89	0.85	0.67		0.89	0.85
2	Fluoxetine	20	20	20	42	81	41		330.68	171.46	192.9	2.1		4.05	2.05
	D-fluoxetine				87	125	98		159.64	111.11	113.84	4.35		6.25	4.9
	D-F/F				2.07	1.54	2.39		0.48	0.65	0.59	2.07		1.54	2.39
3	Fluoxetine	60	60	40	288	420	296		144.67	99.2	93.84	4.8		7	7.4
	D-fluoxetine				133	193	155		313.28	215.88	179.21	2.21		3.21	3.87
	D-F/F				0.46	0.46	0.52		2.17	2.18	1.91	0.46		0.46	0.52
4	Fluoxetine	20	20	20	61	66	61		227.68	210.43	227.68	3.05		3.3	3.05
	D-fluoxetine				144	176	138		96.45	78.91	100.64	7.2		8.8	6.9
	D-F/F				2.36	2.67	2.26		0.42	0.37	0.44	2.36		2.67	2.26
5	Fluoxetine	40	40	20	162	193	51		171.46	143.92	272.33	4.05		4.82	2.55
	D-fluoxetine				295	426	189		94.16	65.2	73.48	7.37		10.65	9.45
	D-F/F				1.82	2.21	3.71		0.55	0.45	0.27	1.82		2.21	3.71
6	Fluoxetine	60	60	60	273	367	361		152.62	113.53	115.42	4.55		6.11	6.01
	D-fluoxetine				132	155	146		315.65	268.81	285.38	2.2		2.58	2.43
	D-F/F				0.48	0.42	0.40		2.07	2.37	2.47	0.48		0.42	0.40
*MEAN* (*SD*)	Fluoxetine	36.66(8.02)	36.66(8.02)	30(6.83)	153.5 (43.53)	208.16 (61.68)	163 (56.19)		207.35 (28.24)	144.32 (16.71)	182.96 (27.53)	3.88 (0.44)		5.23 (0.57)	4.21 (0.85)
	D-fluoxetine				142.5 (33.01)	197.33 (47.46)	144.66 (11.97)		187.56 (41.52)	142.29 (33.24)	152.98 (31.07)	4.42 (0.96)		6.15 (1.28)	5.18 (1.05)
	D-F/F				1.31 (0.35)	1.36 (0.38)	1.68 (0.53)		1.06 (0.33)	1.15 (0.36)	1.08 (0.36)	1.31 (0.35)		1.36 (0.38)	1.68 (0.53)
7	Sertraline	100	100	100	13.3	17.1	11.4		5221.38	4061	6091.61	0.133		0.171	0.114
	Nor-sert				40.4	45.7	37.7		1718.92	1519.57	1842	0.4		0.45	0.37
	N-S/S				3.04	2.67	3.31		0.33	0.37	0.30	3.01		2.63	3.25
8	Duloxetine	90	90	90	46.7	24.7	36.4		1338.33	2530.36	1717	0.51		0.27	0.4
9	Duloxetine	30	30	30	1.5	0.62			13888.88	33602.15		0.05		0.021	
10	Topiramate	100	100	100	2.96	5.53	4.2		23460.96	12557.76	16534.39	0.03		0.055	0.042
11	Aripipazol	5	5	5	31.9	45.2	28		108.84	76.819	124	6.38		9.04	5.6
	D-Aripiprazol				16.8	14.9	12.4		1.89	233.03	280.01	3.36		2.98	2.48
	D-Arip/Arip				0.52	0.32	0.44		206.68	3.03	2.25	0.52		0.32	0.44
12	Clobazam	10	10	10	142	155	136		48.9	44.8	51	1.42		1.55	1.36
	N-clobazam				362	456	551		19.18	15.22	12.60	36.2		45.6	55.1
	N-clob/clob				2.54	2.94	4.05		0.39	0.33	0.24	25.49		29.41	40.51
13	Bupropion	150	150	150	40.7	113.5	24.7		2559.37	917.76	4217.27	0.27		0.75	0.16
	OH-Bupropion				1451	1585	473		71.79	65.72	220.22	9.67		10.56	3.15
	OH-Bup/Bup				35.65	13.96	19.14		0.02	0.07	0.05	35.81		14.08	19.68
14	Bupropion	150	150	150	4	75.8	82		26041.66	1374.23	1270.32	0.02		0.5	0.54
	OH-Bupropion				478	3639	3387		217.92	28.62	30.75	3.18		24.26	22.58
	OH-Bup/Bup				119.5	48	41.3		0.01	0.02	0.02	159		48.52	41.81

*CONC*: Concentration; *CL*: apparent clearance; *CDR*: concentration-dose ratio; *CDRw*: concentration-dose ratio adjusted by weight; *D-F/F*: quotient between D-Fluoxetine and Fluoxetine; *N-S/S*: quotient between Nor-sertraline and Sertraline; *D-Arip/Arip*: quotient between D-Aripiprazol and Aripiprazol; *N-Clob/Clob*: quotient between N-clobazam and Clobazam; *OH-Bup/Bup*: quotient between OH-Bupropion and Bupropion; *SD*: standard deviation

### Fluoxetine (n = 6)

The mean age was 51.3 years (*SD* 2.7). The mean *BMI* of the patients was 38.7 (*SD* 1.8) in M1, 34.5 (*SD* 1.7) in M2 and 28.5 (*SD* 2.2) in M3. Kilograms of total body fat measured by BOD-POD were 51.85 (*SD* 3.5) in M1, 44.4 (*SD* 3.8) in M2 and 28 (*SD* 6.4) in M3.

The mean fluoxetine + D-fluoxetine concentration was 296 (*SD* 60.1) ng/mL in M1, 405.5 (*SD* 81.4) in M2 and 307.66 (*SD* 59.1) in M3. Furthermore, the mean *CDR* of fluoxetine + D-fluoxetine was 8.3 (*SD* 0.8) in M1, 11.39 (*SD* 0.94) in M2, and 9.39 (*SD* 0.82) in M3.

**Fig. 1:**
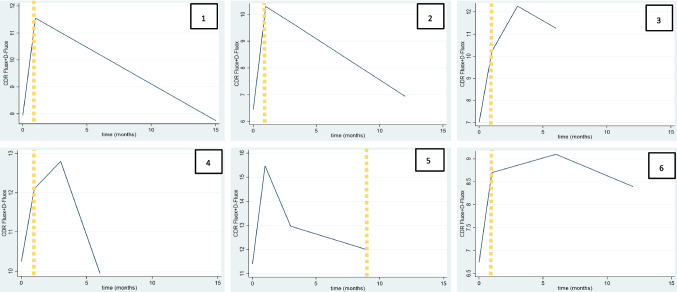
Curve of CDR fluoxetine plus CDR D-fluoxetine throughout time. The yellow line shows the peak of BMI loss

A statistically significant difference was found between the concentrations and the *CDR* of fluoxetine + D-fluoxetine, comparing M1 and M2 (*p* = 0.027) but none between M1 and M3 (*p* = 0.074) or M2 and M3 (*p* = 0.115). Figure 1 shows the change in *CDR* throughout the postoperative period and its relationship with the peak weight loss rate.

**Fig. 2:**
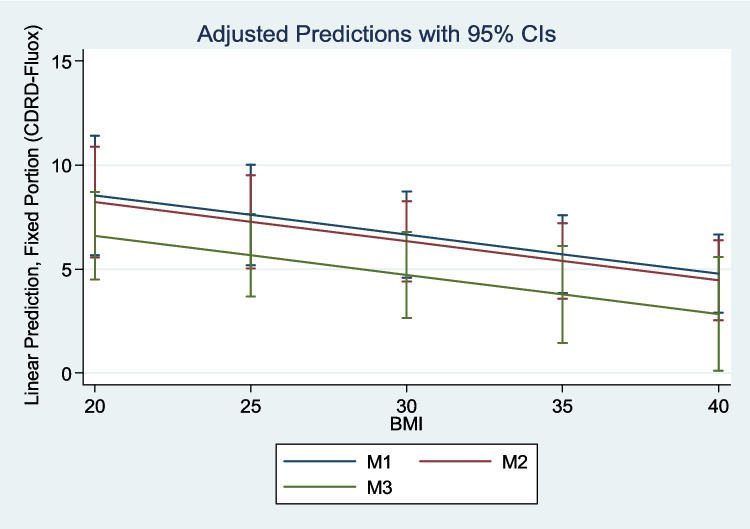
Correlation between concentration-dose ratio of D-fluoxetine (CDR-D-fluox) and body mass index (*BMI*) adjusted by postoperative time with a mixed methods model (*X* axis = *BMI* and *Y* axis = CDR D-fluoxetine)

The mixed-effects regression model revealed a significant negative relationship between *BMI* and *CDR* of D-fluoxetine (coefficient = −0.188, *p* = 0.004). The random-effects analysis showed variability in individual responses to treatment (*SD* of the intercept = 2.224). The Wald chi-square statistic for the model was 8.47 (*p* = 0.014) (Figure 2).

### Other drugs (n = 8)

**Fig. 3:**
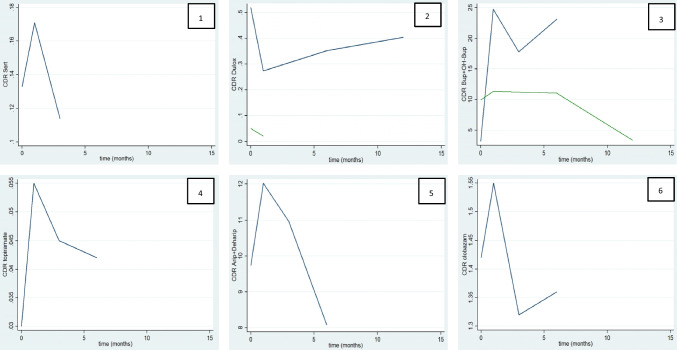
Curve of *CDR* thought time of the other drugs. 1: Sertraline; 2: Duloxetine (blue: patient 8; green: patient 9); 3: Bupropion plus OH- bupropion (blue: patient 13; green: patient 14); 4: Topiramate; 5: Aripiprazole plus D-Aripiprazole; and 6: Clobazam

The pharmacokinetics, as well as the changes in *CDR* after the intervention, in patients receiving treatment with duloxetine (*n* = 2), bupropion (*n* = 2), sertraline (*n* = 1), clobazam (*n* = 1), topiramate (*n* = 1), and aripiprazole (*n* = 1), are shown in Table 2 and Figure 3.

## Discussion

The key observation from this study is that, within the limitations of a small sample size and retrospective design, chronic use of antidepressants did not seem to modify major clinical decisions regarding the management of patients with obesity undergoing MBS. The decision between RYGB vs. sleeve gastrectomy as well as antidepressant dosage should be guided by the patient’s clinical condition. Choosing between these surgical techniques involves multiple factors, such as age, associated medical conditions, and overall surgical eligibility. Postoperative antidepressant dosage needs a careful monitoring of the symptoms to ensure an appropriate therapeutic response. While this observation is exploratory, it provides valuable insight and warrants further investigation through well-designed studies and meta-analyses to confirm these results and their implications for clinical practice.

The results of this study showed that patients within the therapeutic range preoperatively remained within this range post-surgery, both in terms of *CDR* and serum concentration [14, 19–24]. Additionally, our data indicate that there are no significant changes in pharmacokinetic parameters among patients measured at different times between 6 and 15 months post-surgery (M3). This consistency suggests that metabolic stabilization occurs within this period, and the pharmacokinetics of antidepressants remain stable. Importantly, none of the patients exhibited instability in their psychiatric conditions requiring neither admission, nor medication adjustments.

On the other hand, two distinct absorption patterns were identified. The first group (fluoxetine, sertraline, aripiprazole, topiramate, bupropion, and clobazam) exhibited a peak in serum concentration between the first and third postoperative months, followed by a return to baseline. The second pattern (duloxetine) showed a minimum serum concentration at 1 month, before stabilizing. The main difference between duloxetine and the rest lies in its primary metabolic pathway, which is predominantly via CYP2D6, while the others are largely metabolized by CYP3A4 or CYP2C9 [25, 26]. These enzyme locations are mainly hepatic in the case of CYP2D6, while those of CYP3A4 and CYP2C9 can be found in the liver and proximal intestine [25, 26]. Cytochromes P450 (CYPs) are responsible for the majority of phase I reactions in the metabolism of most drugs [25], and this observation led us to hypothesize that the enzymatic pathway could play a crucial role in shaping the pharmacokinetic changes observed postoperatively [27].

Additionally, recent studies have shown in vitro that CYP3A4 is affected by *BMI*, significantly decreasing its function in patients with severe obesity, restoring its function 6 months after surgery, probably because of an upward adjustment of liver metabolism. Furthermore, the negative effect of inflammation on CYP3A4 function must be considered, as demonstrated by studies on the metabolism of certain cytostatics, such as docetaxel, when used in patients with a significant inflammatory burden due to their oncological condition [28–30]. A similar phenomenon occurs with clobazam and CYP2C19 [27, 31–33] as well as with bupropion and CYP2B6 [10, 27, 34, 35]. Meanwhile, CYP2D6 and CYP2C9 functions are independent of body weight [33].

In agreement with these observations, we have confirmed a significant inverse association between *BMI* and the *CDR* of D-fluoxetine, which is the product of CYP3A4 metabolism of fluoxetine. This suggests that metabolic adaptations following weight loss, including CYP3A4 upregulation, play a critical role in drug disposition. Duloxetine, on the other hand, is mainly metabolized by CYP2D6 [33, 36], whose activity remains unaffected by *BMI* or MBS [33]. This difference in metabolic pathways likely explains the distinct pharmacokinetic profiles observed between fluoxetine and duloxetine.

A systematic review by Mass et al. [37] analyzed 12 papers on antidepressant uptake after metabolic bariatric surgery (MBS), revealing lower blood concentrations of sertraline and duloxetine between 2 weeks and 1 year post-surgery [15, 38, 39]. Other drugs, such as paroxetine, escitalopram, bupropion, and fluoxetine, showed inconclusive variations [12, 15, 39, 40]. Studies by Garin et al. and Wallerstedt et al. [12, 15] reported similar behavior for fluoxetine, although without statistical significance, compared to baseline values. Duloxetine showed a decrease in serum concentration during the first month, returning to baseline or lower levels later [12, 15]. The literature on changes in topiramate, sertraline, bupropion, and aripiprazole after MBS is limited, mostly consisting of case reports or small case series [13, 41–43].

Beyond enzymatic metabolism, the anatomical rearrangement following RYGB plays a critical role in drug absorption. Physiological changes include a reduction in gastric mixing, an increase in pouch pH, accelerated gastric emptying, a decrease in surface area available for drug absorption, and altered bile salt entry into the digestive tract, despite their overproduction post-surgery. These factors collectively impact the dissolution, solubility, and subsequent bioavailability of orally administered drugs, particularly those with pH-dependent solubility [11, 44]. Moreover, changes in intestinal transit time and the bypass of key absorption sites, such as the duodenum and proximal jejunum, can further affect the bioavailability of drugs like fluoxetine, which rely on specific segments of the intestine for optimal absorption [33, 35, 45]. Additionally, drug transporters, such as P-glycoprotein (P-gp), which are highly expressed in the small intestine, may also alter drug bioavailability by actively effluxing drugs back into the intestinal lumen. At the intestinal level, catabolic function takes place in the villi of enterocytes and is dependent on their surface area and the amount of substrate accessed [46].

Ultimately, our results indicate that, in this series, chronic use of antidepressants did not require changes to major management decisions after MBS. Two distinct absorption patterns for different antidepressants were noted, which may be influenced by metabolic pathways and enzymatic activity. Despite some early postoperative fluctuations, the long-term pharmacokinetics appeared stable. However, due to the small sample and short follow-up, the continued efficacy and safety of these medications post-surgery should be evaluated further in larger prospective studies.

This study has several important limitations, including the small number of patients and drugs analyzed, the lack of a control group, and its retrospective nature, which may limit the generalizability and strength of the conclusions. Accordingly, our findings should be considered exploratory and hypothesis-generating, rather than definitive.

Studies with a larger number of patients and drugs, with a follow-up of 1 year or more, analyzing the enzymatic facet in each patient, are needed to elucidate those responsible for the phenomena described and the modifications in the CYP complex in the long term.

## Conclusions

This study suggests that, in this limited cohort, chronic use of antidepressants may not impact major decisions in managing MBS patients. It identifies two distinct absorption patterns for different antidepressants post-surgery, highlighting the possible role of metabolic pathways and enzymatic activity. The *CDR* of D-fluoxetine showed an inverse association with *BMI*, which may have its origin in the enzymes belonging to the CYP complex and their behavior after MBS. However, due to the study’s limitations, further research with larger sample sizes and prospective designs is warranted to validate and expand upon these findings.

## Data Availability

In accordance with our commitment to preserve patient privacy and confidentiality, we wish to clarify that research data from this study are only provided upon formal request. Access to the data is strictly controlled and granted only to individuals or organizations with a legitimate need for the information, and who have agreed to comply with all relevant data protection regulations.
